# Aberrant methylation-mediated downregulation of lncRNA *CCND2 AS1* promotes cell proliferation in cervical cancer

**DOI:** 10.1186/s40709-020-00122-5

**Published:** 2020-06-26

**Authors:** Chengcheng Zhao, Jian Liu, Huazhang Wu, Jiaojiao Hu, Jianquan Chen, Jie Chen, Fengchang Qiao

**Affiliations:** 1 Women’s Hospital of Nanjing Medical University, Nanjing Maternity and Child Health Care Hospital, Nanjing, China; 2 Central Laboratory, The Affiliated Jiangning Hospital of Nanjing Medical University, Nanjing, China; 3 Department of Oncology Gynecology, The First Affiliated Hospital of Bengbu Medical College, Bengbu, China; 4 School of Life Sciences, Anhui Province Key Laboratory of Translational Cancer Research, Bengbu Medical College, Bengbu, China

**Keywords:** lncRNA, *CCND2 AS1*, Cell proliferation, DNA methylation, Cervical cancer

## Abstract

**Background:**

Long non-coding RNA (lncRNA) plays an important role in tumorigenesis. The lncRNA *CCND2 AS1* has been shown to be involved in the growth of several tumors; however, its role in cervical cancer has not been elucidated. This study aimed to explore the expression, function, and underlying mechanism of action of *CCND2 AS1* in cervical cancer. Expression of *CCND2 AS1* was examined in cervical cancer and adjacent normal cervical tissues by quantitative real-time polymerase chain reaction (qRT-PCR) and by bioinformatic analysis of data from the Gene Expression Profiling Interactive Analysis (GEPIA) database. The function of *CCND2 AS1* was investigated by overexpressing or silencing *CCND2 AS1* in HeLa and SiHa cervical cancer cells followed by in vitro and in vivo analyses. Methylation-specific PCR (MSP) and bisulfite genomic sequencing (BGS) were used to detect *CCND2 AS1* promoter methylation status in cervical cancer cells.

**Results:**

*CCND2 AS1* expression was lower in cervical cancer compared with normal cervical tissues, and the level was significantly correlated with the patient age and tumor size. *CCND2 AS1* overexpression inhibited the proliferation and cell cycle progression of HeLa cells in vitro and/or in vivo, whereas *CCND2 AS1* silencing had the opposite effects. *CCND2 AS1* expression was elevated after treatment of cervical cancer cells with the DNA methyltransferase inhibitor 5′-azacytidine (5′-Aza), and this was mediated, at least in part, via reduced CpG methylation at the *CCND2 AS1* promoter.

**Conclusion:**

*CCND2 AS1* expression is downregulated in cervical cancer, potentially through increased *CCND2 AS1* promoter methylation, and the upregulation of *CCND2 AS1* expression inhibited tumor growth. These data suggest that *CCND2 AS1* could be a diagnostic marker and potential therapeutic target for cervical cancer.

## Background

Cervical cancer is the second leading cause of cancer-related death in women in developing countries, despite the widespread implementation of screening and treatment programs [1]. If diagnosed at an early stage, cervical cancer can be treated successfully, but patients with advanced stage disease have limited treatment options [2]. The known risk factors for cervical cancer include human papilloma virus infection and genetic aberrations such as somatic mutations in genes such as *KRAS*, *PIK3CA*, *PTEN*, *STK11*, and *TP53* [3–6], and copy number variations in other genes [7, 8]. Despite an increase in studies of the molecular mechanisms involved in cervical carcinogenesis, our understanding of the events remains incomplete. Thus, there is an urgent need to identify the molecules and mechanisms that contribute to cervical cancer, both to uncover novel diagnostic markers and to identify potential therapeutic targets.

Long non-coding RNAs (lncRNAs) were previously thought to be “transcriptional noise” but are now recognized to play crucial roles in physiological and pathological processes. LncRNAs are defined as RNA sequences of > 200 nucleotides in length that have no or limited protein-coding capacity. LncRNAs located in the nucleus and cytoplasm have different functions; thus, in the nucleus, lncRNAs regulate gene expression at the level of transcription and mRNA splicing, whereas in the cytoplasm, lncRNAs affect mRNA stability and regulate protein function [9]. The mechanisms by which lncRNAs act are also varied, and include chromatin modulation, DNA binding, and RNA processing [10]. In normal and transformed cells, lncRNAs play pivotal roles in proliferation, migration, invasion, differentiation, and apoptosis [11, 12]. For example, Wu et al. showed that *lincRNA*-*p21*, a transcriptional target of p53, binds to the E3 ubiquitin-protein ligase MDM2 and feeds back to enhance p53 transcriptional activity [13]. In turn, p53 interacts with p300 and binds to the promoters/enhancers of *lincRNA*-*p21*. Therefore, *lincRNA*-*p21* was identified as a novel regulator of cell proliferation and apoptosis and has been suggested as a therapeutic target for atherosclerosis and related cardiovascular disorders [13]. LncRNA *GClnc1* acts as a scaffold to recruit the WDR5 and KAT2A methyltransferase complex proteins, which modify the transcription of various target genes that affects the proliferation, invasiveness, and metastasis of gastric cancer cells [14]. LncRNA *MALAT1* promotes the proliferation of CasSki cervical cancer cells by diminishing expression of the cell-cycle regulatory proteins cyclin D1, cyclin E, and CDK6 [15]. In cervical cancer, the lncRNA *MALAT1* regulates apoptosis by influencing the expression of caspase 3, caspase 8, Bcl2, Bax and Bcl-xL, and additionally enhances cell invasion and metastasis by upregulating the expression of Snail and affecting the epithelial–mesenchymal transition [16]. Moreover, lncRNA *CCND2 AS1* has been shown to be significantly overexpressed in papillary thyroid carcinoma cell lines, and its knockdown significantly suppressed cell proliferation, migration, and invasion in vitro [17]. In glioma, *CCND2 AS1* promotes proliferation and growth via effects on Wnt/β-catenin signaling [18]. However, little is known about the expression and/or function of lncRNA *CCND2 AS1* in cervical cancer.

In the present study, we investigated *CCND2 AS1* expression in fresh cervical tissues and in GEPIA datasets, and probed its function by overexpressing or silencing *CCND2 AS1* in cervical cancer cell lines and analyzing the effects in vitro and in a mouse xenograft model. Furthermore, we examined the mechanisms underlying altered *CCND2 AS1* expression and function by examining the methylation status of the *CCND2 AS1* promoter in cervical cancer.

## Methods

### Tissue samples

A total of 46 pairs of cervical cancer tissue and nearby non-tumor tissue were collected from patients at the First Affiliated Hospital of Bengbu Medical College, China, during 2016 and 2017. This study was approved by the Ethic Committee of the Obstetrics and Gynecology Hospital Affiliated to Nanjing Medical University and patients provided informed consent. After excision, all tissues were immediately frozen in liquid nitrogen until RNA extraction.

### Bioinformatic analysis

The expression level of *CCND2 AS1* in tissues was analyzed using Gene Expression Profiling Interactive Analysis (GEPIA), a web server for cancer and normal gene expression profiling and interactive analyses [19]. The |Log_2_FC| cutoff was 1 and the *p*-value cutoff was 0.01. A box plot was generated to show the differential expression of *CCND2 AS1* between 306 cervical cancer tissues and 13 normal cervical tissues.

### Quantitative real-time PCR (qRT-PCR)

RNA was extracted from 100 mg of tissue samples or 5 × 10^6^ cells using Trizol reagent (Invitrogen, USA) as previous described [20]. The quality and quantity of the RNA were assessed by agarose gel electrophoresis and NanoDrop spectrophotometry (ND-1000) (Thermo Fisher Scientific, Waltham, MA, USA). RNA was reverse transcribed to cDNA using PrimeScript™ RT reagent Kit with gDNA Eraser (TAKARA, Japan) according to the manufacturer’s instruction. LncRNA expression levels were examined using a SYBR Green PCR kit (TAKARA, Japan). Primers were synthesized by Genscript Corporation (Nanjing, China). Glyceraldehyde 3-phosphate dehydrogenase (GAPDH) was analyzed as an internal control for normalization. The primer sequences were (5′ to 3′): *CCND2 AS1* forward CAAGCTGGAACCCTGCAAGA, reverse AAGGGTATACCTTCCTCCCCAA; GAPDH forward AGAAGGCTGGGGCTCATTTG, and reverse GCAGGAGGCATTGCTGATGAT. The amplification conditions were 95 °C for 30 s, followed by 40 cycles of 95 °C for 30 s and 60 °C for 30 s. Relative lncRNA expression levels were quantified by the 2^−ΔΔCt^ method.

### Cell culture and transfection

The human cervical cancer cell lines HeLa and SiHa were obtained from the cell bank of the Chinese Academy of Science (Shanghai, China) and were cultured in DMEM medium (Wisent, China) with 10% fetal bovine serum (FBS, Wisent) and 1% penicillin and streptomycin at 37 °C in a humidified 5% CO_2_ incubator. *CCND2 AS1* and a control scramble sequence were synthesized by Genscript and cloned into pcDNA3.1 vector to produce pCCND2 AS1 and pcDNA3.1 plasmids, respectively. For transfection, HeLa and SiHa were plated at 1.5 × 10^5^ and 2 × 10^5^ cells per well, respectively, in a 6-well plate for 24 h, and then transfected with pCCND2 AS1 or pcDNA3.1 using Lipofectamine 2000 (Invitrogen, Carlsbad, CA, USA) following the manufacturer’s instructions. G418 (Gibco, Grand Island, USA) was added at 400 μg μl^−1^ to generate the stable HeLa and SiHa cell lines. HeLa and SiHa cells were also transiently transfected with small interfering RNAs (siRNAs; GenePharma, China) against *CCND2 AS1* and negative control (NC) sequences. The sequence were (5′ to 3′): SiRNA1 sense GGGCUGGUCUCUUUGAGUUTT, antisense AACUCAAAGAGACCAGCCCTT; SiRNA2 sense: GCCAAGAAACGGUCCAGAATT, antisense UUCUGGACCGUUUCUUGGCTT; SiRNA3 sense GCAAAUCUGAAGCCACAAATT, antisense AACUCAAAGAGACCAGCCCTT.

### Cell proliferation assay

Cell proliferation was detected using a colorimetric Cell Counting kit-8 (CCK-8; Dojindo Molecular Technologies, Kumamoto, Japan) following the manufacturer’s protocol. The kit contains WST-8 [2-(2-methoxy-4-nitrophenyl)-3(4-nitrophenyl) -5-(2,4-disulfophenyl)-2H-tetrazolium, monosodium salt], which produces a water-soluble formazan dye upon reduction in the presence of an electron mediator, as is reduced by dehydrogenases in cells to give an orange colored product (formazan), which is soluble in the culture medium. The amount of the formazan dye generated by dehydrogenases in cells is directly proportional to the number of living cells. Cells were added to 96-well plates at 1 × 10^3^ 100 μl^−1^ per well (n = 3) and cultured for 1, 2, 3, 4, 5 or 6 and 7 days. The medium was then replaced with a mixture of 90 μl of fresh DMEM medium and 10 μl of CCK‑8 reagent and the cells were incubated for an additional 2 h. The absorbance at 450 nm, which is proportional to cell number, was measured using a microplate reader (iMark, USA).

### Cell cycle

Cells were cultured at 1 × 10^6^ well^−1^ in 6-well plates and synchronized by starvation by culturing in medium with 1% FBS for 2 days. The cells were then fixed in cold 70% alcohol at −20 °C, washed twice with phosphate-buffered saline (PBS) containing 1% FBS, and then treated with PBS containing 0.02% TritonX-100, 0.1 mg ml^−1^ RNase (Sigma-Aldrich, St. Louis, Missouri, USA), and 10 mg ml^−1^ propidium iodide (Sigma-Aldrich, St. Louis, Missouri, USA) for 30 min at 37 °C. Cell cycle distribution was examined by flow cytometry using a FACScan flow cytometer (Becton–Dickinson, San Jose, CA, USA). The relative number of cells in each phase of the cell cycle was analyzed using the Modfit program (Verity Software House, Topsham, ME, USA) [21].

### Tumor xenograft mouse model

Female BALB/c nude mice at 4-week-old were purchased from Laboratory Animal Center of Yangzhou University. For each cell type, 10^7^ cells in 100 μl PBS were injected into the right dorsal flank of mice. Tumor size was monitored weekly using a Vernier caliper to measure the length (L) and width (W) of the tumors. Volume was calculated as: V = 0.5 × L × W^2^. The mice were euthanized 4 weeks after injection and the tumors were removed and weighed. The experiments were conducted in accordance with the guidelines of the Obstetrics and Gynecology Hospital Affiliated to Nanjing Medical University.

### Western blot analysis

At 72 h after transfection, aliquots of 5 × 10^6^ cells were lysed in RIPA buffer (Beyotime Institute of Biotechnology, China), and the lysate was determined using a BCA Protein Assay Kit (Beyotime, Shanghai, China). A total of 30 μg protein per lane was resolved by 10% SDS-PAGE and transferred to a PVDF membrane (Merck Millipore, USA). The membrane was blocked with 5% non-fat milk and then incubated overnight with primary antibodies against β-actin (Sigma-Aldrich, St. Louis, Missouri, USA), CDK-4 (Cell Signaling Technology, USA), CCND1 (Cell Signaling Technology, USA), or CCND2 (Cell Signaling Technology, USA). The membrane was washed in TBST and incubated with the secondary goat anti-mouse IgG antibody (Beyotime Institute of Biotechnology, Shanghai, China). An enhanced chemiluminescence kit (Pierce, Rockford, IL, USA) was used to detect the protein bands using a FluorChem E System (ProteinSimple, CA, USA).

### Methylation-specific PCR (MSP) and bisulfite genomic sequencing (BGS)

Genomic DNA was extracted from cells using an Axygen Miniprep kit and then treated with bisulfite using a Methylamp DNA Modification Kit (Epigentek, USA) according to the manufacturer’s instruction. Samples of 50 ng modified DNA were amplified to determine the proportion of methylated (M) and unmethylated (U) DNA in the promoter region of *CCND2 AS1*. The primer sequences (5′ to 3′) for MSP were: *CCND2 AS1* (M) forward TATAGTTTTTTCGCGGTTAGC, reverse TAAAATCCCGACTCCGAA; *CCND2 AS1* (U) forward ATGTATAGTTTTTTTGTGGTTAGT, and reverse AACTAAAATCCCAACTCCAAA. For BGS, the primers were *CCND2 AS1* forward GTATTTAGGAGTTGTAGATGGG, and reverse CCCCAAACATTTTT TCCAATTAT. The PCR conditions were 95 °C for 30 s, followed by 40 cycles of 95 °C for 30 s and 60 °C for 30 s. PCR products were analyzed by agarose gel electrophoresis. For BGS, PCR products were purified and subcloned into a pEASY-T3 vector (TransGen Biotech, Beijing, China). Ten colonies were randomly chosen and sequenced to assess methylation at each CpG site.

### Statistical analysis

Data were expressed as the mean ± SD of at least three independent experiments. Analyses were performed using SPSS Statistics 17 (SPSS, Chicago, IL, USA). Differences between group means were assessed using Student’s t-test, and the associations between *CCND2 AS1* expression and clinicopathological features were analyzed using the Chi squared (χ^2^) test. All *p* values were two-sided and *p* < 0.05 was considered to be statistically significant.

## Results

### Expression of *CCND2AS1* is decreased in cervical cancer tissues

**Fig. 1:**
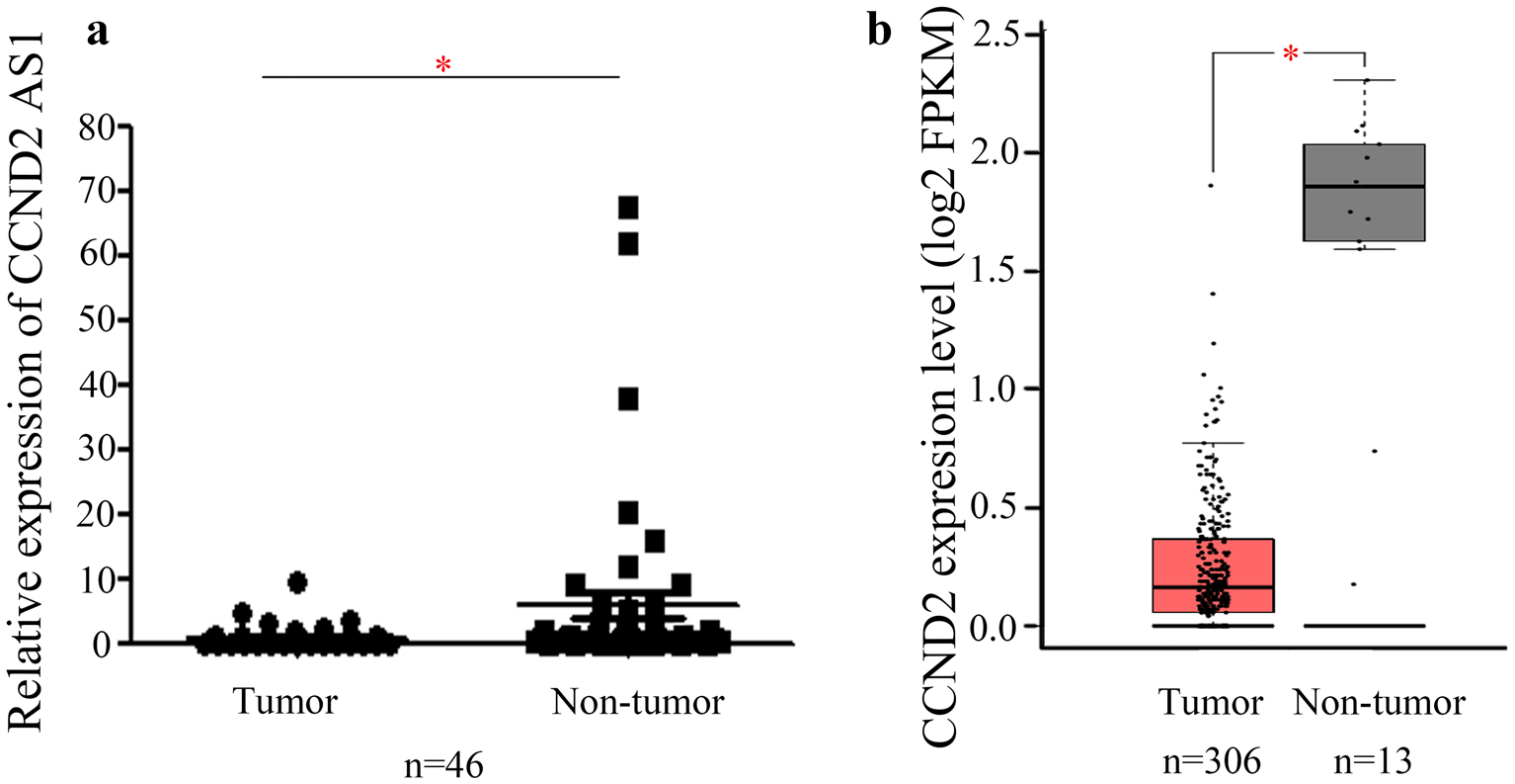
Decreased expression of the long non-coding RNA *CCND2 AS1* in cervical cancer tissues. **a** The expression of *CCND2 AS1* was significant down-regulated in cervical cancer tissues compared with the corresponding adjacent non-tumors tissues (n = 46). The expression of *CCND2 AS1* was detected by qRT-PCR and *GAPDH* expression was used as a reference gene, the black lines are the mean ± SEM (*p < 0.05). **b** The relative expression of *CCND2 AS1* was significant decreased in 306 cervical cancer tissues compared with 13 normal tissues, which was from the GEPIA database. *p < 0.05 (unpaired Student’s t-test)

To investigate the expression pattern of *CCND2 AS1* in cervical cancer tissues, we performed qRT-PCR analysis of 46 pairs of cervical cancer tissues and nearby non-tumor tissues. We found that *CCND2 AS1* levels were significantly lower in the cervical cancer tissues compared with the normal tissues (*p* < 0.05, Fig. 1a). To verify these findings, we also analyzed 306 cervical cancer tissues and 13 normal cervical tissues in a GEPIA dataset consisting of high-throughput sequencing and microarray date for protein-coding and non-coding genes. Consistent with our qRT-PCR results, we found that *CCND2 AS1* was significantly lower in the cervical cancer tissues compared with the 13 normal tissues (*p* < 0.05, Fig. 1b). Next, we explored the associations between *CCND2 AS1* expression and patient clinicopathological characteristics in the 46 cervical cancer patients. As shown in Table 1, low *CCND2 AS1* expression was significantly associated with patient age (*p *= 0.029) and tumor size (*p *= 0.033), suggesting a potential role for *CCND2 AS1* in cervical cancer growth.

**Table 1:** Relationship between CCND2 AS1 expression in cervical cancer patients and clinicopathologic characteristics

Variable	Number	CCND2 AS1 expression		*p* value
		Low	Moderate/high	
Age	44			
≥ 50		11	7	0.029
< 50		14	2	
Tumor size	44			
≥ 4 cm		14	7	0.033
< 4 cm		7	16	
Histology	43			
Squamous cell carcinoma		21	15	0.971
Adenocarcinoma		4	3	
Lymph node metastasis	44			
Yes		11	4	0.199
No		14	15	
FIGO stage	44			
Ib–IIa		13	10	1
IIb–IIIa		12	9	

### Overexpression of *CCND2 AS1* inhibits the proliferation of cervical cancer cells by inducing G1/S phase arrest

**Fig. 2:**
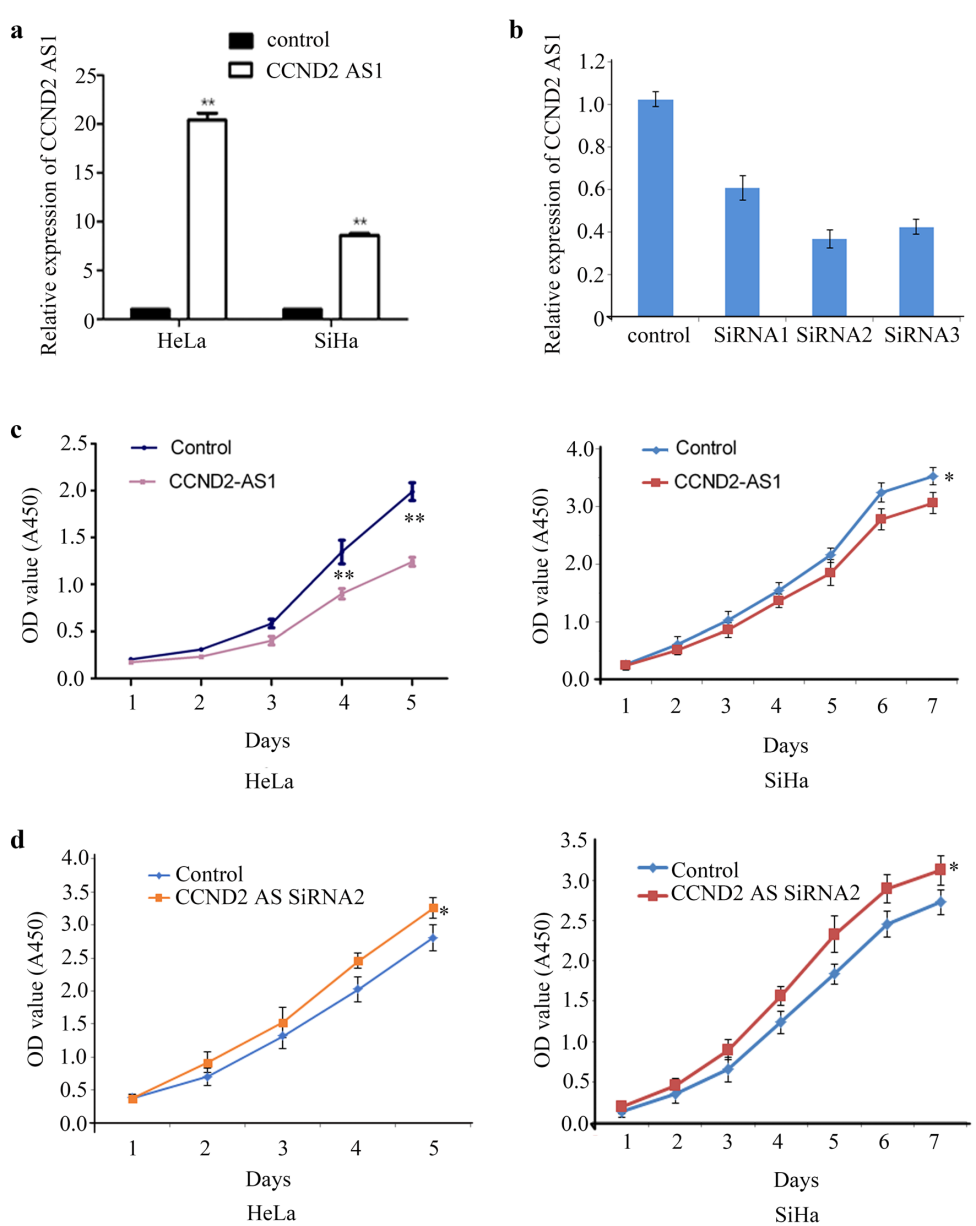
*CCND2 AS1* regulates cell proliferation in cervical cancer cell lines. **a** The *CCND2 AS1* overexpression efficiency was detected by qRT-PCR in human HeLa and SiHa cell lines. **b** The knockdown efficiency of *CCND2 AS1* SiRNAs in HeLa and SiHa cell lines were validated by qRT-PCR. **c** Enforced expression of *CCND2 AS1* inhibited cell proliferation in HeLa and SiHa cell lines. *p < 0.05, **p < 0.01. **d** Knockdown of *CCND2 AS1* expression by siRNA2 promoted cell proliferation in HeLa and SiHa cell lines. *p < 0.05

To investigate the functions of *CCND2 AS1*, we overexpressed or knocked down *CCND2 AS1* in the human cervical cancer cell lines HeLa and SiHa, both of which express very low endogenous levels of *CCND2 AS1* (Fig. 2a). As expected, qRT-PCR analysis of cells transfected with a *CCND2 AS1* expression vector indicated a significant increase in expression compared with cells transfected with the control plasmid (Fig. 2a). Conversely, transfection of HeLa cells with three different *CCND2 AS1* siRNAs significantly reduced *CCND2 AS1* expression (Fig. 2b). Based on its superior ability to reduce *CCND2 AS1* expression, siRNA2 was selected for the remaining experiments.

**Fig. 3:**
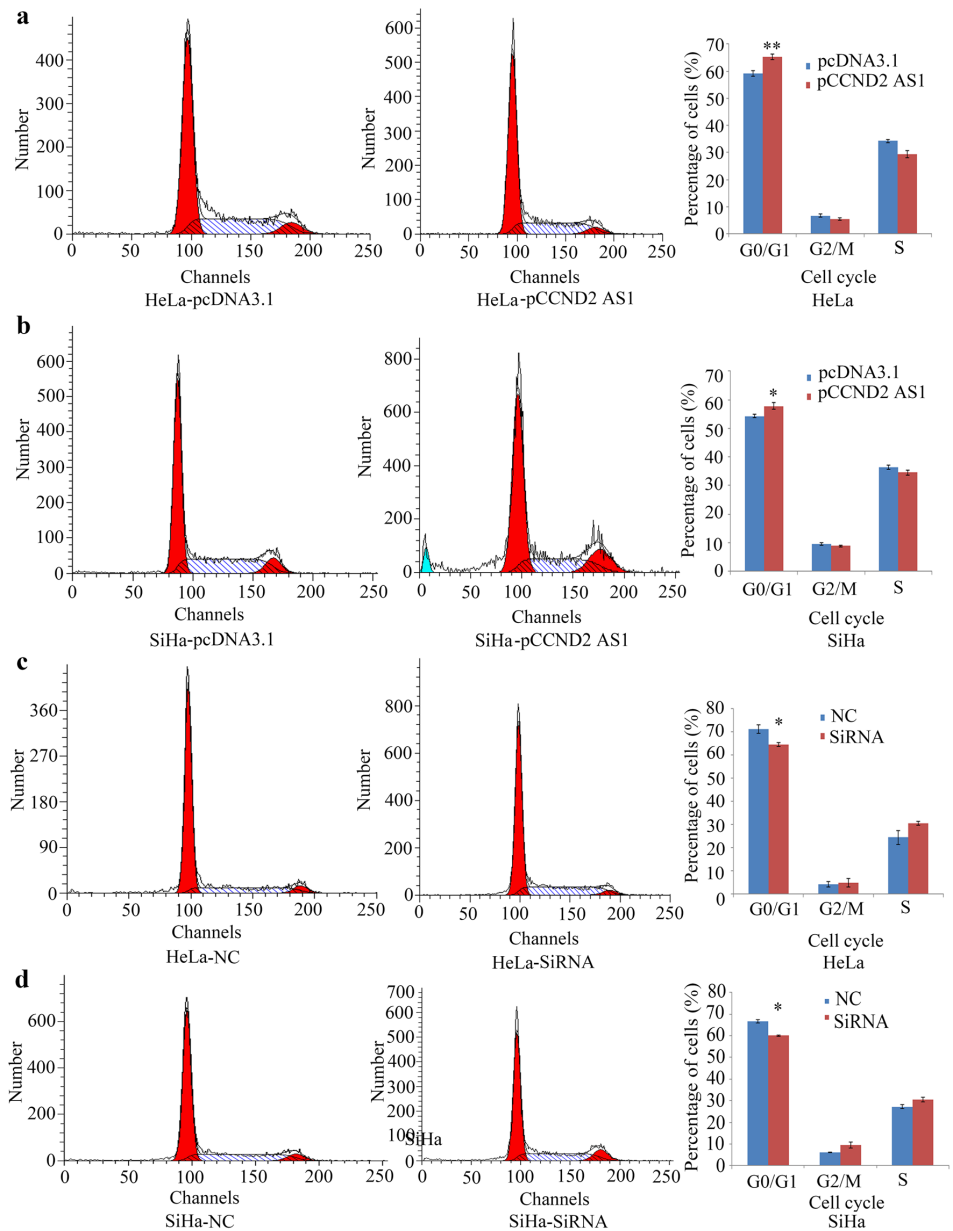
*CCND2 AS1* inhibits cell proliferation by arresting the cell cycle at the G1/S phase in cervical cancer cell lines. **a**, **b** The distribution of cell cycle phases analysis by flow cytometry between enforced expression of pCCND2 AS1 and control (pcDNA3.1) in HeLa and SiHa cell lines. The percentages of cells in the G1, S, and G2/M phases are shown in the bar chart as the mean ± SD of three independent experiments. *p < 0.05, **p < 0.01. **c**, **d** Flow cytometry analysis of the distribution of cell cycle phases in negative control (NC) and *CCND2 AS1* SiRNA2 in HeLa and SiHa cell lines. The percentages of cells in the G1, S, and G2/M phases are shown in the bar chart as the mean ± SD of three independent experiments. *p < 0.05

Overexpression of *CCND2 AS1* had different effects on the proliferation of HeLa and SiHa cells, as assessed using the CCK-8 assay. Thus, overexpression of *CCND2 AS1* significantly inhibited HeLa cell proliferation in vitro on days 4 and 5. In SiHa cell line, cells transfected with *CCND2 AS1* grew slower than control cells at day 7 (Fig. 2c). However, in both HeLa and SiHa cells, proliferation was enhanced by siRNA-mediated silencing of *CCND2 AS1* (Fig. 2d). To investigate the mechanism by which *CCND2 AS1* affects proliferation, the cell cycle progression of HeLa and SiHa cells overexpressing *CCND2 AS1* was analyzed using propidium iodide staining of DNA followed by flow cytometry. As shown in Fig. 3a, b, the percentage of cells in G1 phase of the cell cycle was significantly higher in cells overexpressing *CCND2 AS1* compared with those expressing the negative control (NC) sequence in both HeLa cells (64.51% *vs* 59.23%) and SiHa cells (58.42% *vs* 54.32%). Conversely, the percentages of cells in G1 phase were significantly slower in HeLa-pCCND2 SiRNA2 (64.56%)/SiHa-pCCND2 SiRNA2 (59.65%) compared to HeLa-NC (71.23%) and SiHa-NC cells (67.35%) (Fig. 3c, d). These data suggest that elevated levels of *CCND2 AS1* induce arrest of cervical cancer cells at the G1/S phase.

### Overexpression of *CCND2 AS1* inhibits cervical cancer cell proliferation by suppressing CDK4, CCND1, and CCND2 expression

**Fig. 4:**
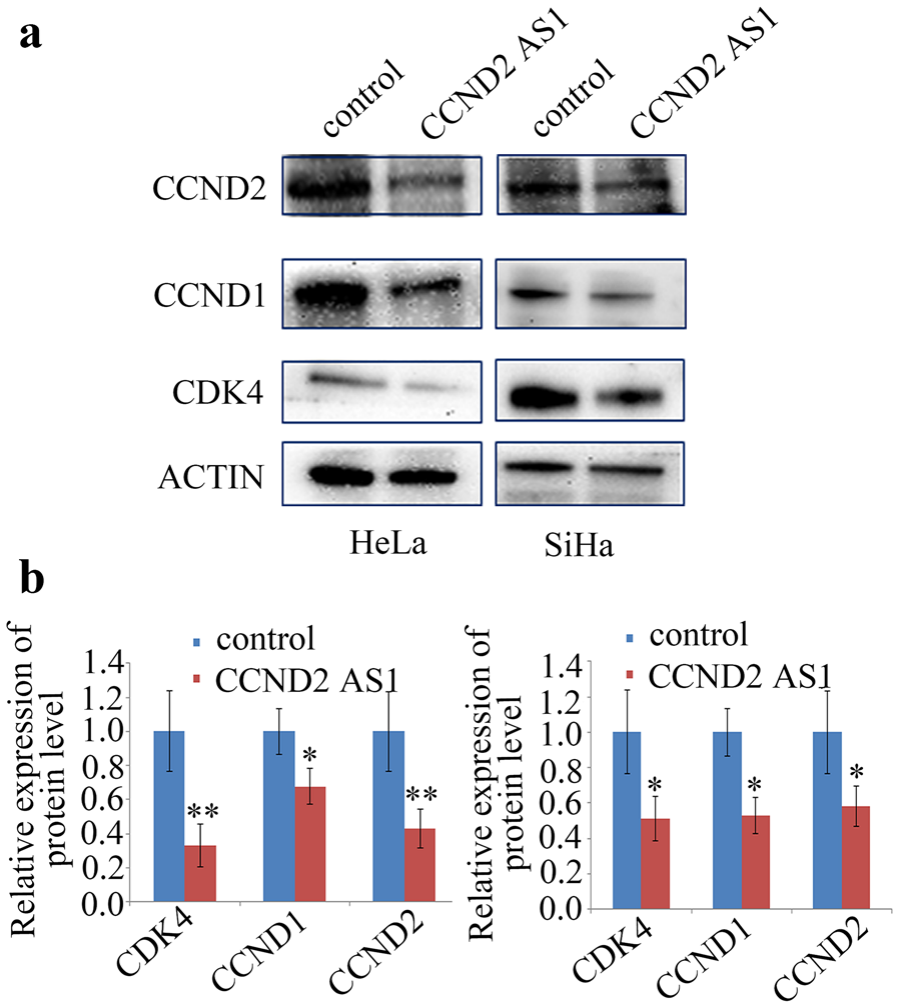
Enhanced expression of *CCND2 AS1* reduced CCND1, CCND2 and CDK4 expression. **a** CCND1, CCND2 and CDK4 expression detected by western blot in HeLa and SiHa cell lines. **b** Densitometry of CCND1, CCND2 and CDK4 protein expression of three independent experiments. *p < 0.05, **p < 0.01

Next, we explored the mechanism of *CCND2 AS1* inhibition of the cell cycle by measuring expression of the cell-cycle regulatory proteins CCND2, CCND1, and CDK4. Western blot analysis followed by quantification of the protein levels indicated that CCND2, CCND1, and CDK4 proteins were significantly downregulated in HeLa and SiHa cells transfected with the *CCND2 AS1* overexpression plasmid compared with the control plasmid (Fig. 4a, b).

### Overexpression of *CCND2 AS1* suppresses cervical cancer cells tumorigenesis in vivo

**Fig. 5:**
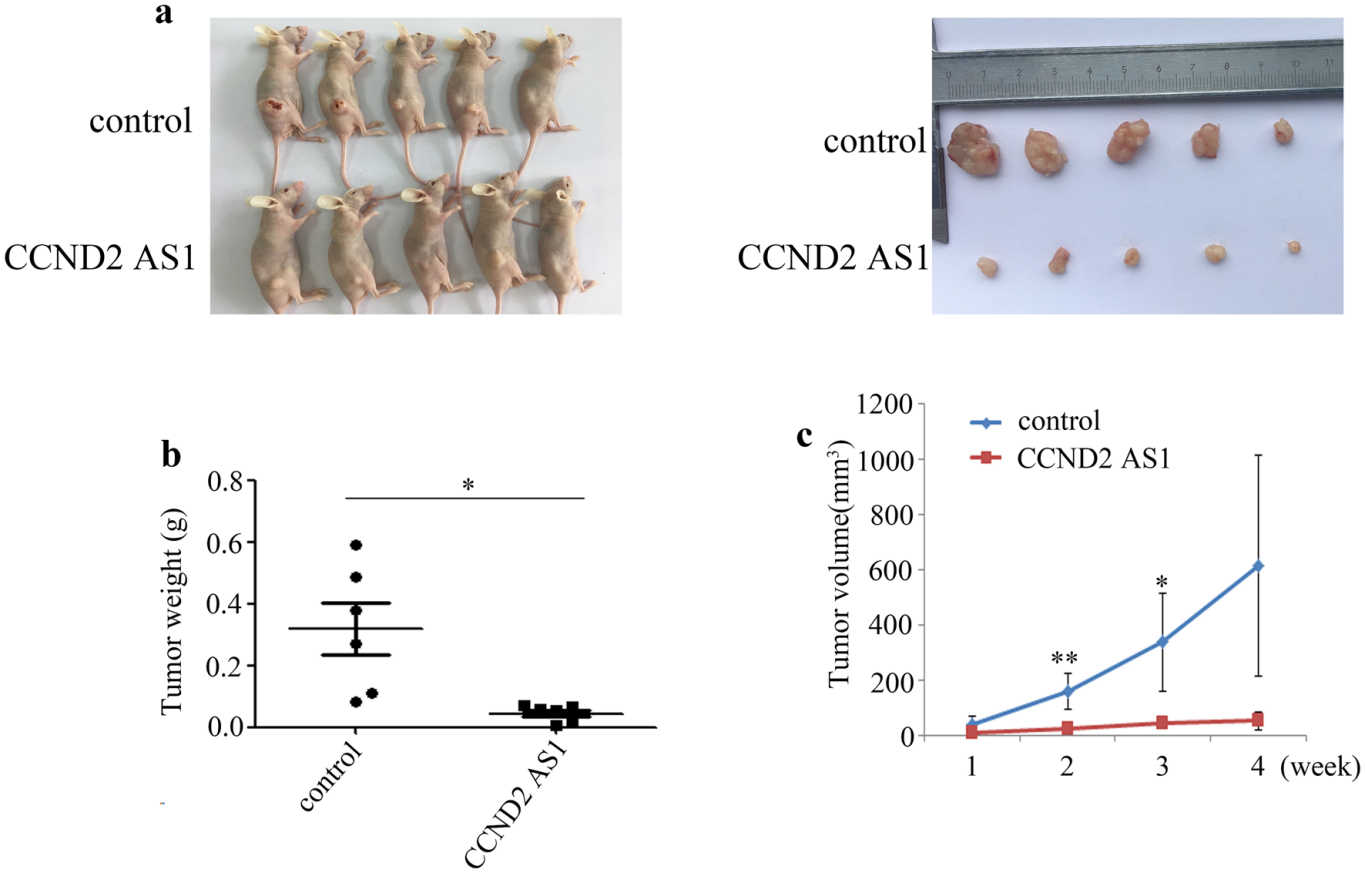
Elevated expression of *CCND2 AS1* suppressed tumor growth in vivo. **a** Images of tumors formed in the nude mice injected with the indicated cells. The control or HeLa-pCCND2 AS1 were injected into the right flanks (n = 5), respectively. **b**, **c***CCND2 AS1* up-regulated significantly inhibited tumor growth in tumor weights and sizes in nude mice. Tumor volumes were calculated by the formula: V = 0.5 × L × W^2^ after injection every week. Bars indicate SD. Asterisk(s) indicates a significant change (*p < 0.05, **p < 0.01). Data are the mean ± SD

We assessed the tumor suppressor effect of *CCND2 AS1* overexpression in vivo using a mouse xenograft model. For this analysis, groups of BALB/c nude mice (n = 10) were subcutaneously injected with HeLa cells transfected with the *CCND2 AS1* overexpression plasmid or a control plasmid, and tumor growth was monitored. Solid tumors were visible in all mice within 4 weeks of injection (Fig. 5a). However, compared with the control animals, mice overexpressing *CCND2 AS1* exhibited significantly reduced tumor growth, as reflected by the tumor weights and volumes (Fig. 5b, c). These data confirm the in vitro analyses by demonstrating that *CCND2 AS1* upregulation inhibits cervical cancer growth in vivo.

### Silencing of *CCND2 AS1*expression due to its promoter CpG methylation in cervical cancer

**Fig. 6:**
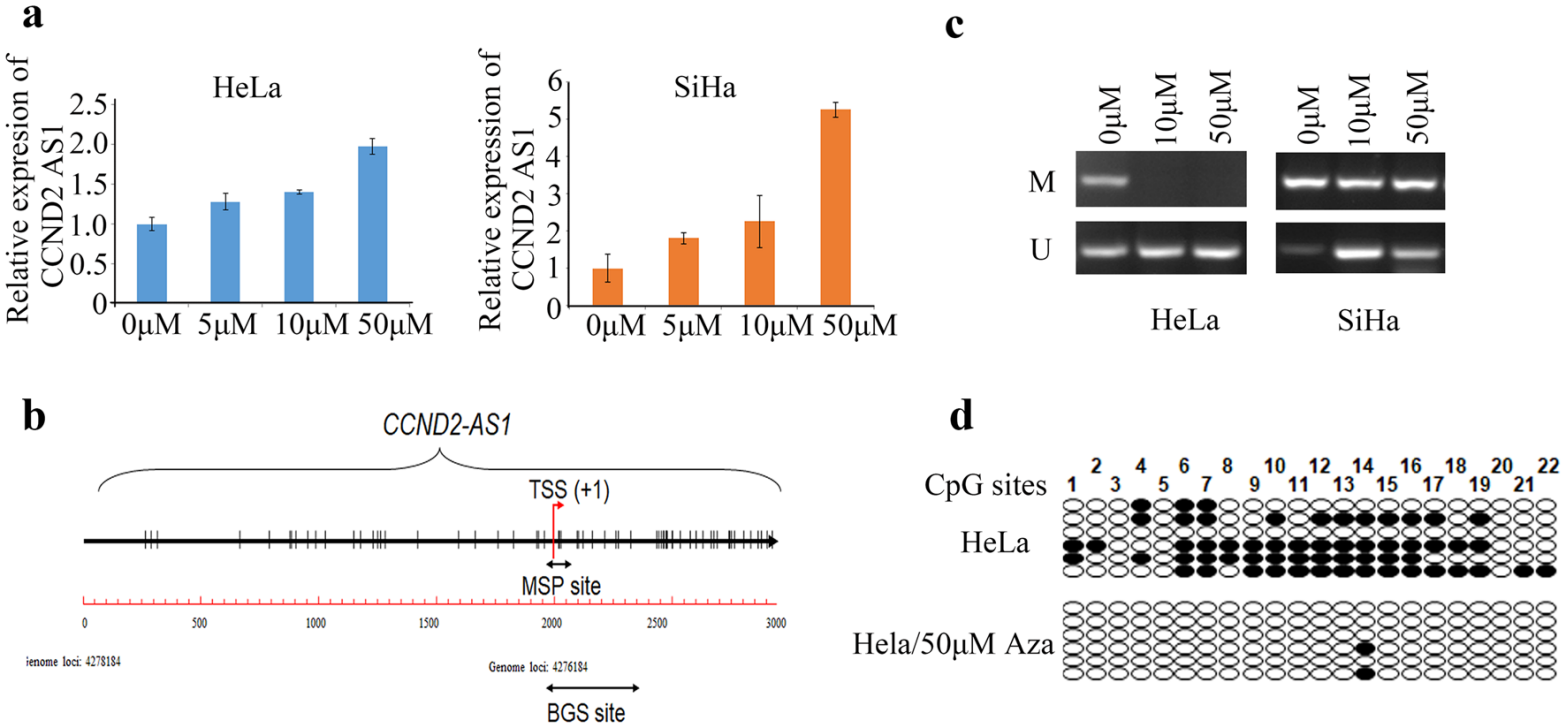
Silencing of *CCND2 AS1* due to its promoter CpG methylation in cervical cancer. **a***CCND2 AS1* expression was restored when the HeLa and SiHa cells were treated with 5΄-Aza from 5 μM to 50 μM. **b** Diagram of the *CCND2 AS1* gene promoter with the transcription start site (TSS) indicated. The short black double-headed arrow represents the location of the fragment detected by the MSP and BGS assay. **c** Methylation status of the *CCND2 AS1* promoter was detected by Methylation-specific PCR in HeLa and SiHa cell lines. M showed methylation-specific primer amplification, and U showed unmethylation-specific amplification. **d** Bisulfite genomic sequencing analysis was performed spanning the *CCND2 AS1* core promoter and exon 1 in HeLa cell line. Twenty-two individual CpG sites within the *CCND2 AS1* promoter were sequenced. Each row represents a single sequence; white circle indicates unmethylated CpG sites and black circle indicates methylated CpG sites

To investigate whether the low endogenous expression level of *CCND2 AS1* in HeLa and SiHa cells is regulated by the gene methylation status, we treated the cells with the DNA methyltransferase inhibitor 5′-azacytidine (5′-Aza) and examined *CCND2 AS1* expression by qRT-PCR. Notably, *CCND2 AS1* expression was dose-dependently increased in both cell types within 72 h of 5′-Aza treatment (Fig. 6a). Next, we examined whether DNA methylation of the *CCND2 AS1* promoter directly contributed to its low endogenous level by performing methylation-specific PCR (MSP). After treatment with 50 μM of 5′-Aza for 72 h, the proportion of methylated to unmethylated promoter sequence decreased in HeLa cells, and in SiHa cells, more sites were transformed to unmethylated status, although this was less consistent compared to HeLa cells (Fig. 6b, c).Thus, the *CCND2 AS1* promoter was highly methylated in HeLa and SiHa cells in the basal state. Next, we performed bisulfite genomic sequencing (BGS) analysis of CpG sites in the *CCND2 AS1* core promoter and exon 1 of HeLa cells. Methylated CpG sites were readily detected in untreated HeLa cells, but they were essentially absent from 5′-Aza-treated cells, which confirmed the results of the MSP analysis (Fig. 6d).

## Discussion

In this study, we show for the first time that lncRNA *CCND2 AS1* expression is downregulated in cervical cancer tissue compared with adjacent normal tissue. This was demonstrated by direct analysis of patient samples as well as by bioinformatic analysis of a GEPIA dataset. Decreased *CCND2 AS1* expression correlated with patient age and tumor size, pointing to a potential role for *CCND2 AS1* in cervical cancer cell growth. We confirmed this to be the case by examining cervical cancer cell lines in vitro, which revealed that *CCND2 AS1* overexpression or knockdown led to inhibition or promotion, respectively, of cell proliferation. We demonstrated that *CCND2 AS1* regulates proliferation, at least in part, via effects on the cell-cycle regulatory proteins CCND1, CCND2, and CDK4. Thus, overexpression or knockdown of *CCND2 AS1* caused a significant accumulation or reduction, respectively, of cervical cancer cells at the G1/S phase, indicating a block at the G1/S transition. Finally, we verified the in vivo relevance of the in vitro findings by confirming that the growth of cervical cancer tumors was significantly reduced by upregulation of *CCND2 AS1*.

Our results differ from those observed in other types of cancer. Xia et al. found that *CCND2 AS1* knockdown significantly suppressed the proliferation, migration, and invasion of the TPC1 thyroid cancer cell line, whereas overexpression had the opposite effects [17]. The same responses to manipulation of *CCND2 AS1* expression were observed with the BCPAP thyroid cancer cell line [17]. *CCND2 AS1* may have contributed to these effects through regulation of N-cadherin and vimentin expression [17]. Zhang et al. showed that *CCND2 AS1* promoted glioma cell proliferation and growth by enhancing Wnt/β-catenin signaling [18]. Collectively, these data suggest that the molecular events regulated by *CCND2 AS1* may vary depending on the cancer type. Further investigations of *CCND2 AS1* expression and function in additional cancers will be necessary to confirm these observations.

DNA methylation is one of the most important mechanisms of epigenetic regulation of lncRNA expression, and aberrant methylation may contribute to carcinogenesis. Yang et al. showed that lncRNA *GAS5* expression was decreased in parallel with increased *GAS5* methylation in cervical cancer cells, and forced overexpression of *GAS5* inhibited the growth and metastatic behavior of the cells [22]. Pang et al. identified the landscape of tumor suppressor lncRNAs in 33 breast cancer specimens using whole transcriptome sequencing, and they validated the results with a TCGA dataset [23]. These authors identified multiple lncRNAs that were negatively regulated at the transcriptional level by epigenetic modifications such as DNA methylation and histone modification [23]. He et al. found that lncRNA *AFAP1*-*AS1* expression correlated negatively with the promoter CpG methylation status in both lung cancer cells and patient tissues, and the DNA methyltransferase inhibitor decitabine significantly increased *AFAP1*-*AS1* expression [24]. Similarly, in the present study, we demonstrated that *CCND2 AS1* expression was upregulated by treatment with the demethylation reagent 5′-Aza in cervical cell lines, suggesting that *CCND2 AS1* transcription might also be regulated via promoter methylation/demethylation in this type of cancer. Indeed, MSP and BGS indicated a negative correlation between *CCND2 AS1* expression and the abundance of methylated CpG sites in HeLa cells. In SiHa cells, methylation of the *CCND2 AS1* promoter was not significantly changed by 5′-Aza, but the abundance of the unmethylated *CCND2 AS1* promoter was increased significantly. These results indicate that *CCND2 AS1* expression in cervical cancer cells is partly regulated by CpG methylation of the gene promoter, supporting promoter methylation as a common mechanism for regulating lncRNA expression in tumors.

## Conclusions

This is the first report that *CCND2 AS1* acts as a tumor suppressor in cervical cancer. We found that decreased expression of *CCND2 AS1* was associated with patient age and tumor size, and forced overexpression *CCND2 AS1* inhibited cervical cancer cell proliferation in vitro and in vivo, at least partly by arresting the cells at the G1/S phase. Low *CCND2 AS1* expression likely results from enhanced methylation of the gene promoter in these cells. Collectively, our data suggest that *CCND2 AS1* could be a novel diagnostic marker and/or a potential therapeutic target for cervical cancer. However, further work will be required to verify these findings and to elucidate in more detail the molecular mechanisms by which *CCND2 AS1* regulates the growth of cervical cancer.

## Data Availability

All data generated or analyzed during this study are included in this published article.

## Abbreviations

RT-qPCR: Quantitative real-time PCR
MSP: Methylation-specific PCR
BGS: Bisulfite genomic sequencing
